# Association between Salt-Related Knowledge, Attitudes, and Behaviours and 24 h Urinary Salt Excretion in Nepal

**DOI:** 10.3390/nu16121928

**Published:** 2024-06-18

**Authors:** Kamal Ghimire, Shiva Raj Mishra, Dinesh Neupane, Per Kallestrup, Craig S. McLachlan

**Affiliations:** 1 Nepal Development Society, Bharatpur-10, Chitwan 44200, Nepal; shivrajmishra@gmail.com (S.R.M.); neupane.dinesh@gmail.com (D.N.); 2 School of Health, Torrens University, Sydney, NSW 2010, Australia; 3 NHMRC Clinical Trials Centre, Faculty of Medicine and Public Health, The University of Sydney, Camperdown, NSW 2050, Australia; 4 Department of International Health, Johns Hopkins Bloomberg School of Public Health, Johns Hopkins University, Baltimore, MD 21212, USA; 5 Research Unit for Global Health, Department of Public Health, Aarhus University, 8000 Aarhus, Denmark; per.kallestrup@ph.au.dk; 6 Centre for Healthy Futures, Torrens University, Sydney, NSW 2010, Australia; reperfusion@hotmail.com

**Keywords:** salt, sodium, 24 h urine, hypertension, blood pressure, knowledge, attitude, behaviours, community based, Nepal

## Abstract

This study examined the association between salt-related knowledge, attitudes, and behaviors (KAB) and salt excretion using the 24-hour (24 h) urinary collection method. Data were utilized from the Community-Based Management of Non-Communicable Diseases in Nepal (COBIN) Salt Survey, a community-based cross-sectional study conducted among a sub-sample of COBIN cohort in Pokhara Metropolitan City, Western Nepal, from July to December 2018, among adults aged 25–70 years. A total of 451 adults participated in the study, and a single 24 h urine sample was collected from each participant. The mean [(standard deviation (SD)] age of the participants was 49.6 (9.82) years, and the majority were female (65%). The mean urinary salt excretion was 13.28 (SD: 4.72) g/day, with 98% of participants consuming ≥5 g of salt/day. Although 83% of participants knew the risks of high salt intake and 87% believed it was important to reduce their intake, only 10% reported doing so. Salt-related attitude i.e., self-perceived salt intake was significantly associated with urinary salt excretion, adding extra salt to food, consuming processed foods, and taking actions to salt control. Participants who perceived themselves as consuming high salt had higher urinary salt excretion [(14.42 g/day; 95% confidence interval (95% CI): 13.45, 15.39, *p* = 0.03)], were more likely to add extra [(Odds ratio (OR) = 3.59; 95% CI: 2.03, 6.33, *p* < 0.001)], and consume processed foods more often (OR = 1.90; 95% CI: 1.06, 3.40, *p* < 0.05) compared to those who self-perceived consuming a normal amount of salt. Conversely, participants who perceived themselves as consuming low salt were more likely to take actions to control salt intake (OR = 4.22; 95% CI: 1.90, 9.37, *p* < 0.001) compared to their counterparts who perceived consuming a normal amount of salt. There existed a gap between salt-related knowledge, attitudes, and actual behaviors, resulting in a high salt intake among the Nepalese population. Nepal urgently requires tailored national salt reduction programs that comprise both policy and community-level interventions to achieve a 30% reduction in mean population salt intake by 2025. Further validation studies are needed to assess the effectiveness of community-based intervention in Nepal.

## 1. Introduction

Salt (sodium chloride) is composed of sodium (Na^+^) and chloride (Cl^−^) ions. Sodium (Na) is an essential element for living beings to maintain normal cell function, nerve impulse transmission, and many other functions [1]. Excessive sodium intake, however, is correlated with negative health outcomes such as increased blood pressure (BP) and hypertension and related cardiovascular diseases (CVDs) [2,3]. The recent Global Burden of Disease Study 2019 estimated that coronary heart disease and stroke ranked second and third among the top ten leading causes of death and disability combined in Nepal [4]. High BP and unhealthy diets were among the top five risk factors for this death and disability [4]. Reducing dietary sodium intake at the population level is one of the most feasible and cost-effective public health interventions for preventing CVDs [5].

Estimates from both global and local studies suggest that the Nepalese population has much higher dietary salt consumption [6,7] than the World Health Organization’s (WHO’s) recommended level of <5 g/day [1]. Global modelling in 2010 estimated the mean salt intake in Nepal to be ~10 g (3.89 sodium)/day [6], and the average salt intake derived from available Nepalese studies ranged from 8 to 14.4 g/day [7]. However, no specific salt reduction program or activities have been initiated in the country to address this high consumption [7,8].

The identification of sources of dietary salt and the exploration of individuals’ knowledge, attitudes, and behaviours pertaining to salt intake represent a critical avenue of inquiry for illuminating the underlying drivers of excessive salt consumption [9]. Evidence suggests that enhanced salt-related knowledge and favourable attitudes towards reducing salt intake may serve as catalysts for behavioural modifications, ultimately resulting in decreased salt consumption [10,11,12]. However, despite this evidence, there remains a paucity of nationally representative data and studies examining these factors in Nepal. The earlier findings of this dataset reported that the Nepalese population had a much higher salt intake, measured at 13.28 g/day using the 24-hour (24 h) urinary excretion method [13]. Alternative methods, such as the spot urine method, were noted to have limitations for estimating 24 h salt intake in Nepal [14]. The primary objective of the present study was to assess the association between salt-related knowledge, attitudes, and behaviours (KAB) and 24 h urinary salt excretion. The secondary objective was to explore the association between salt-related knowledge and attitudes and salt-related behaviours. The findings of this study can add to the knowledge base to design tailored salt reduction programs and activities in Nepal.

## 2. Materials and Methods

### 2.1. Study Design and Settings

This study utilized data from the Community-Based Management of Non-Communicable Diseases in Nepal (COBIN) Salt Survey, a community-based cross-sectional study conducted in Pokhara Metropolitan City (formerly known as Lekhnath Municipality) in Western Nepal among adults from July to December 2018 [13]. The detailed methodology of the COBIN Salt Survey was published previously [13]. In brief, the subsample for the COBIN Salt Survey was selected from the original COBIN project cohort, which was initiated in 2013 and recruited 2815 participants from Pokhara Municipality using the WHO standard formula for the Stepwise Approach to Non-Communicable Disease Risk Factor Surveillance (STEPS) survey [15]. The study area is a hilly urban setting with a total population of 513,504 in 2021 [16]. While many households adhere to traditional home-cooked meals and dining practices, out-of-home dining at restaurants and street vendors has become increasingly popular in this area. The inclusion criteria required participants to be enrolled in the COBIN cohort and to provide consent for participation in this study. Participants were excluded if they were pregnant or menstruating at the time of urine collection. However, in instances where participants were menstruating, they were revisited after one week for urine collection.

### 2.2. Sample Size Calculations

This study used systematic random sampling to select 500 participants out of the 2815 enrolled in the COBIN cohort. The sample size for this study was determined using the Cochran formula, where the sample size was calculated at a 96% CI (Z = 1.96) and a 5% margin of error [17]. Salt estimation is an under-studied area in Nepal. No studies have provided the required prevalence figure for sample size calculation; therefore, we used a maximum variability of 50% as the prevalence fraction, which resulted in a sample size of 385.

**Figure 1:**
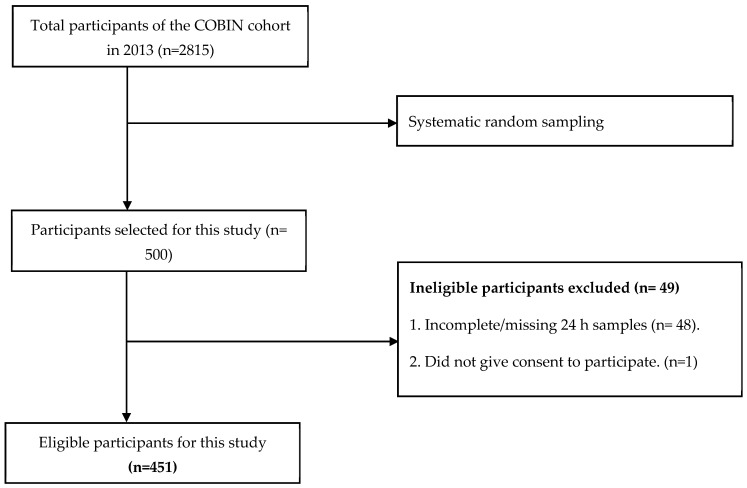
Flowchart of the study participants.

Furthermore, since the study population is finite and small (2815), we used the modified version of the Cochran formula in the next step to derive the required sample [17]. The required sample size for this study was 338 based on the modified formula. However, in order to account for the higher likelihood of a non-response, urine sample wastages, and under-representation of demographic subgroups, we conveniently oversampled the population until a maximum cap of 500 was reached. A total of 49 samples were excluded from further analysis as they were ineligible because of missing 24 h samples (48) and non-consent (1), resulting in a sample size of 451 (Figure 1).

### 2.3. Measurements

#### 2.3.1. Salt-Related Knowledge, Attitudes, and Behaviours

The salt-related KAB of participants were collected using a standardized WHO STEPS questionnaire [18]. There was one knowledge-related question, two attitude-related questions, and three major behaviour-related questions. There were seven additional questions related to regular actions to control salt intake. Some of the response options of KAB were modified/merged because of very low/low observations in some categories, thereby simplifying analyses and interpretation. Details of the salt-related KAB questionnaire are given in the Supplementary Files (Table S1).

#### 2.3.2. Twenty-Four-Hour Urine Collection

A single 24 h urine sample was collected from each participant. Full instructions (both verbal and written) were given to the participants about the procedure of urine collection for the 24 h period. The instruction was to discard the first void urine in the morning and record the time, then collect all successive urine voids up until the first void of the following morning in the provided jar and record the time of the final urine void. They were instructed to re-collect urine in the case of spillage or a missed collection. The participants were instructed to store the collected urine in a cold, dry place in the provided container with the lid tightly on. On the completion day, field staff collected the urine samples from participants’ homes and transferred them to a local laboratory. Total urine volume and concentrations were measured using standard methods. The concentrations of sodium ions (Na⁺) and potassium ions (K⁺) were determined using the SENSA CORE ST-200 Pro Electrolyte Analyzer(Sensa Core, Hyderabad, India) by an ion-selective electrode (ISE), while creatinine concentration (Cr) was measured using the Gesan Chem 200 Analyzer (Gesan Production, Trapani, Italy) with the Creatinine LR liquid reagent method. Based on the criteria of <500 mL volume of urine, Cr excretion < 6 mmol for men or <4 mmol for women, and self-reported spillage of urine > 30 mL during the 24 h period, 48 urine samples were found incomplete and removed from further analyses. The 24 h urinary sodium content was obtained by multiplying the concentration by the urine volume. The initial Na^+^ was measured in mmol/L which was then converted to mg/day by multiplying by the molar mass of Na^+^, i.e., 23 g/mol. The Na^+^ value was then converted to salt (NaCl) by multiplying by 2.54 (1 mg sodium = 2.54 mg NaCl). Salt (mg/day) was then converted to g/day (by dividing by 1000) to be used in the analyses.

#### 2.3.3. Socio-Demographic Characteristics (Covariates)

Sex, age, caste, education, family size, counselling by health professionals to reduce dietary salt intake, body mass index (BMI), hypertension status, history of elevated BP, antihypertensive drug use, and history of diabetes were used as covariates in the present study. Family size was calculated from the average number of people in a household who consumed morning and evening meals and then dichotomized into small families (≤4 people) and large families (>4 people) based on a median value of 4. BMI was classified as underweight (BMI < 18.5 kg/m^2^), normal (18.5–24.9 kg/m^2^), overweight (25.0–29.9 kg/m^2^), and obese (≥30 kg/m^2^) [19]. Hypertension status was classified as normotension: mean systolic BP (SBP) < 120 mm Hg and diastolic BP (DBP) < 80 mm Hg, prehypertension: mean SBP ≥ 120–139 mm Hg and DBP ≥ 80–<89 mm Hg, hypertension: mean SBP ≥ 140 mm Hg and/or a DBP ≥ 90 mm Hg and/or treatment with antihypertensive drug within 2 weeks [20].

### 2.4. Statistical Analysis

Categorical data were expressed as counts (n) and percentages (%), and continuous data were expressed as mean (standard deviation (SD)). Chi-square tests (or Fisher’s exact tests as appropriate) and analysis of variance (ANOVA) were used to determine the differences in salt-related KAB and 24 h salt excretion, respectively, by socio-demographic characteristics. A general linear model [Analysis of Covariance (ANCOVA)] was used to assess the associations between salt-related KAB and 24 h urinary salt excretion adjusting for possible confounders. Based on the previous literature and the observed association with outcome variables in univariate analyses, age, sex, education, and BMI were considered the confounding variables. Furthermore, binary logistic regression was modelled to examine the association between salt-related knowledge and attitudes and salt-related behaviours. Both crude and adjusted odds ratios together with 95% confidence intervals (95% CI) were computed. The model was adjusted for socio-demographic variables that demonstrated significance with salt-related KAB at a level of *p* < 0.05 in the bivariate analyses. In the bivariate analyses, complete separation was observed between salt-related attitudes (i.e., self-perceived salt intake) and salt-related behaviours (i.e., taking actions to control salt) because of zero observations in some cells. Therefore, the penalized likelihood estimation method was employed for the regression analysis between these variables. Post hoc analyses were conducted using the Bonferroni correction for both ANCOVA and logistic regression models to examine significant associations among group comparisons. Specifically, comparisons were made for groups that showed significant associations in the initial analysis using the *margins*, *pwcompare*, *and mcompare(bonferroni)* commands in Stata. Visualizations were also generated to illustrate the predicted probabilities and Bonferroni-adjusted comparisons across different levels of the categorical variable, providing a clear depiction of the differences among the groups. All data were collated and analysed using STATA 17 for Windows (StataCorp LLC, College Station, TX, USA). All analyses were two-tailed, and *p*-values < 0.05 were deemed significant.

### 2.5. Ethical Approval

This study was approved by the Nepal Health Research Council, Kathmandu, Nepal, under registration number 337/2018, June 2018. All study participants were informed about this study’s objectives and procedures and their role in this study, and written informed consents were obtained.

## 3. Results

### 3.1. Socio-Demographic Characteristics

Of the 451 participants, the majority were female (65%), belonged to the upper caste (74%), and had completed only the primary level of education (59%). Mean (SD) age, BMI, SBP, and DBP were 49.6 (9.82) years, 26.15 (4.28), 129.06 (18.03) mm Hg, and 83.37 (10.26) mm Hg, respectively (Table 1). Nearly one-fifth (18%) and another two-fifths (39%) were obese and hypertensive, respectively (Table 2 and Table 3).

### 3.2. Mean 24 h Urinary Salt Excretion

Per capita mean (SD) 24 h salt excretion was 13.28 (4.72) g/day, which was more than double the WHO recommendation of ≤5 g salt/day [1]. The proportion of participants exceeding the recommendation level was 98% (Table 1). Male participants compared with female participants (14.40 (5.06) versus 12.69 (4.43) g/day)), younger participants compared with older participants (14.34 (4.91) versus 12.73 (4.53) g/day)), and participants with higher compared to lower education (14.08 (3.16) versus 12.70 (4.74) g/day)) all had significantly higher salt intake (*p* < 0.01) (Table 2).

### 3.3. Association between Socio-Demographic Characteristics and Salt-Related Knowledge Attitudes and Behaviours (Bivariate Analysis)

#### 3.3.1. Association with Salt-Related Knowledge and Attitudes

**Figure 2:**
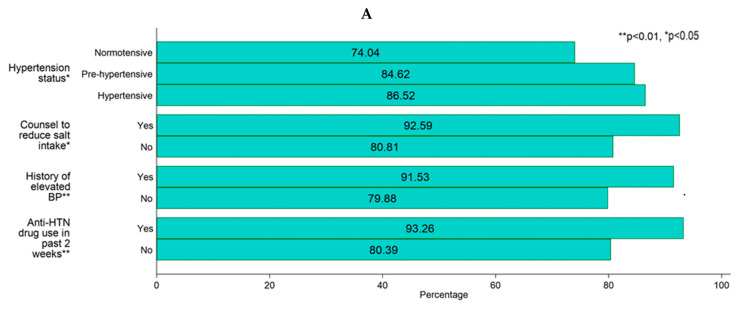

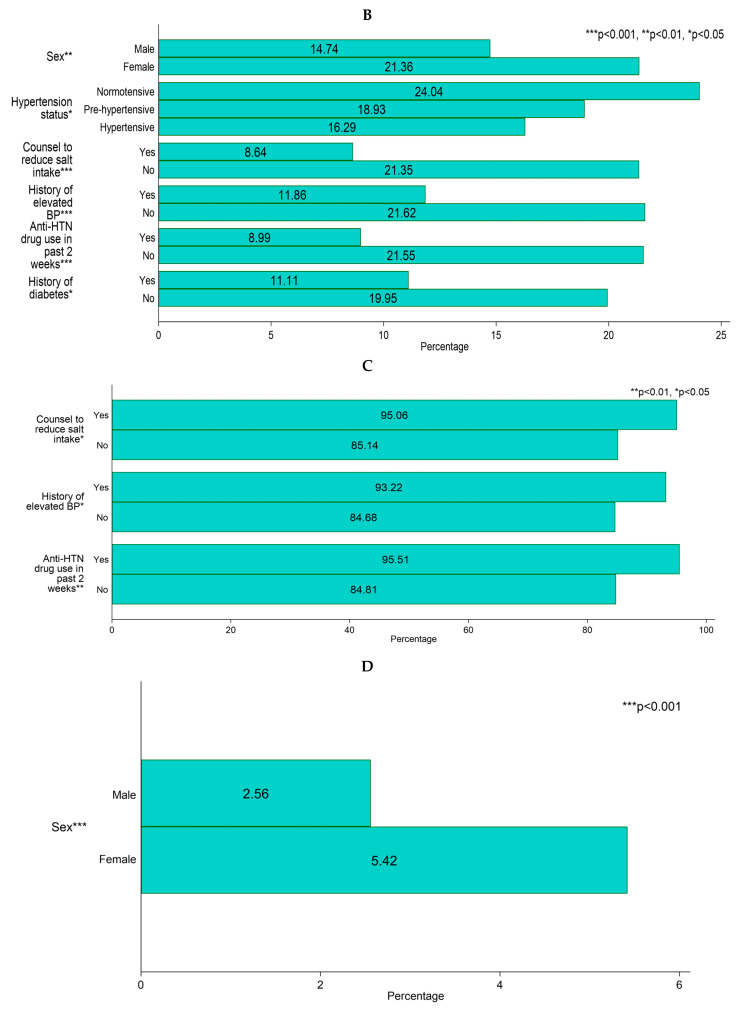

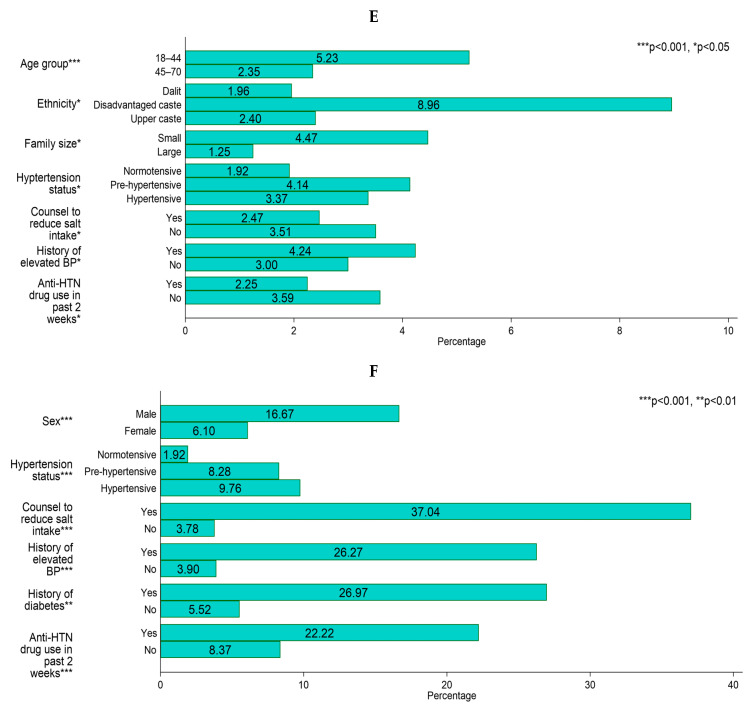
Percentage of participants with salt-related KAB in relation to socio-demographic characteristics: (**A**) salt-related knowledge—yes; (**B**) salt-related attitudes: self-perceived salt intake—consuming extremely high/high salt; (**C**) salt-related attitudes: the importance of lowering salt intake; (**D**) salt-related behaviours—add extra salt; (**E**) salt-related behaviours—processed food consumption; and (**F**) salt-related behaviours—take actions to control salt intake.

A high proportion of participants (83%) were aware that high salt intake causes health problems. The participants’ hypertension status (*p* < 0.05), counselling by a health professional to reduce dietary salt (*p* < 0.05), antihypertensive drug use in the past 2 weeks (*p* < 0.01), and history of elevated BP (*p* < 0.01) were associated with salt-related knowledge. More than half (55%) and nearly one-fifth (19%) of the participants self-perceived consuming normal and extremely high/high salt, respectively. Self-perceived salt consumption was associated with sex (*p* < 0.01), hypertension status (*p* < 0.05), counselling by a health professional to reduce dietary salt (*p* < 0.001), antihypertensive drug use in the past 2 weeks (*p* < 0.001), history of elevated BP (*p* < 0.001), and history of diabetes (*p* < 0.05). Similarly, most (87%) of the participants felt it was very important/somewhat important to reduce salt in their food, which was associated with counselling by a health professional to reduce dietary salt (*p* < 0.05), a history of elevated BP (*p* < 0.05), and antihypertensive drug use in the past 2 weeks (*p* < 0.01) (Table 2, Figure 2).

#### 3.3.2. Association with Salt-Related Behaviours

Only 4% of the participants mentioned their always/often habit of adding extra salt to food before eating, and sex was significantly associated with adding extra salt (*p* < 0.001). Processed food consumption always/often was also not very common in the study participants as only 3% reported doing so. This behaviour was associated with age (*p* < 0.001), caste (*p* < 0.05), family size (*p* < 0.05), hypertension status (*p* < 0.05), counselling by a health professional to reduce dietary salt (*p* < 0.05), history of evaluated BP (*p* < 0.05), and antihypertensive drug use in the past 2 weeks (*p* < 0.05). Only one in ten participants reported taking regular actions to reduce their salt intake. Of those, the majority were engaged in avoiding adding extra salt (82%), avoiding processed food consumption (32%), and checking sodium labels on food (25%). Sex (*p* < 0.001), hypertension status (*p* < 0.001), counselling by a health professional to reduce dietary salt (*p* < 0.001), history of elevated BP (*p* < 0.001), antihypertensive drug use in the past 2 weeks (*p* < 0.001), and diabetes (*p* < 0.01) were associated with actions taken to control salt intake (Table 4, Figure 2).

### 3.4. Association between Socio-Demographic Characteristics and Salt-Related Knowledge, Attitudes, and Salt-Related Behaviours (Logistic Regression)

The odds of adding extra salt to food were more likely among females compared with males (OR = 2.23; 95% CI: 1.22, 4.08, *p* < 0.01), and among those who self-perceived consuming extremely high/high salt (OR = 3.59; 95% CI: 2.03, 6.33, *p* < 0.001), compared with their counterparts (Table 4 and Table S2).

Consumption of processed foods was less likely among the older participants (OR = 0.48; 95% CI: 0.29, 0.79, *p* < 0.01) and those who self-perceived consuming very low/low salt (OR = 0.60; 95% CI: 0.36, 0.98, *p* < 0.05) compared with the younger participants and those who self-perceived consuming extremely high/high salt, respectively. Conversely, the participants from large families (OR = 1.58; 95% CI: 1.01, 2.49, *p* < 0.05) and those who self-perceived consuming extremely high/high salt (OR = 1.90; 95% CI: 1.06, 3.40, *p* < 0.05) were more likely to consume processed foods always/often/sometimes compared with their counterparts (Table 4 and Table S2).

The odds of actions to control salt were more likely among those who were counselled by health professionals to reduce salt intake (OR = 6.89; 95% CI: 2.50, 19.01, *p* < 0.001), and among those who self-perceived consuming very low/low salt (OR = 4.22; 95% CI: 1.90, 9.37, *p* < 0.001) compared with their respective counterparts (Table 4 and Table S2).

Post hoc analyses were conducted to further evaluate the group comparisons of self-perceived salt consumption with two dependent variables as follows: adding extra salt always and taking actions to control salt. For the dependent variable of adding extra salt always, significant differences were observed between the normal and extremely high/high categories (mean difference = −0.26; 95% CI: −0.40, −0.11; *p* < 0.000) and between the very low/low and extremely high/high categories (mean difference = −0.34; 95% CI: −0.50, −0.19; *p* < 0.000). However, no significant difference was found between the very low/low versus normal categories (mean difference = −0.89; 95% CI: −0.19, 0.01; *p* = 0.08). Similarly, for the dependent variable of taking actions to control salt, a significant difference was found between the very low/low and normal categories (mean difference = 1.44; 95% CI: 0.47, 2.41; *p* = 0.001), whereas no significant differences were detected between the normal and extremely high/high categories (mean difference = 1.91; 95% CI: −1.59, 5.41; *p* = 0.57) or the very low/low and extremely high/high categories (mean difference = 3.35; 95% CI: −0.11, 6.82; *p* = 0.06). The post hoc analysis figures are presented in the Supplementary Files (Figures S1 and S2).

### 3.5. Association between Salt Related Knowledge, Attitudes, and Behaviours and 24 h Urinary Salt Excretion (General Linear Model)

**Figure 3:**
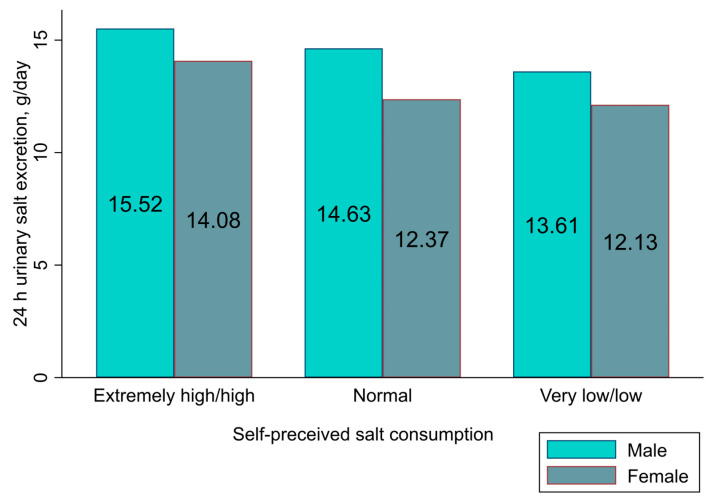
Correlation between self-perceived salt consumption and actual 24 h urinary salt excretion.

There was no significant association between salt-related knowledge (i.e., a high salt diet causes health problems), attitudes (i.e., the importance of lowering salt intake), or behaviours (i.e., adding extra salt, processed food consumption, taking actions to control salt) and 24 h urinary salt excretion. Another attitude (i.e., self-perceived salt consumption), on the other hand, was positively correlated with 24 h urinary salt excretion, such that those who self-perceived that they consumed extremely high/high salt had higher salt excretion than those who perceived themselves consuming very low/low in the actual 24 h urine estimates (14.42 g/day; 95% CI: 13.45, 15.39 versus 12.73 g/day; 95% CI: 11.89, 13.65; *p* = 0.03). The results remained the same after adjusting for possible confounding effects of sex, age, education, and BMI in the general linear model (Table 5, Figure 3). Furthermore, post hoc analyses were conducted using the Bonferroni test for group comparisons. There was a significant difference between the very low/low and extremely high/high categories (mean difference = −1.69; 95% CI: −3.28, −0.10; *p* = 0.03). However, there was no significant difference between the normal and extremely high/high categories (mean difference = −1.31; 95% CI: −2.68, 0.06; *p* = 0.07) or between the very low/low and normal categories (mean difference = −0.38; 95% CI: −1.62, 0.86; *p* = 1.00). The post hoc analysis figures are presented in the Supplementary Files (Figure S3).

## 4. Discussion

This study aimed to investigate the relationship between participants’ salt-related KAB and their 24 h urinary salt excretion. The findings revealed that males, younger participants, those with higher education, and those who perceived themselves as consuming extremely high/high salt had higher salt intake, as reflected in their 24 h urinary estimates. Similarly, individuals in the latter category were also more likely to add extra salt to food and consume processed foods frequently and were less likely to adopt salt reduction behaviours. This places them in a high-risk group for excessive salt consumption. Therefore, salt-related attitudes, such as self-perceived salt consumption, played a significant role in predicting actual salt intake and related behaviours.

The salt-related KAB findings of this study are in accordance with the recent STEPS survey of Nepal [21], which showed that most of the participants had knowledge of the harmful effects of excessive salt intake (83% in the present study and 70.9% in STEPS), had positive attitudes towards the importance of lowering salt in their food (87% and 79.5%), and self-perceived that they consumed a normal amount of salt (55% and 74.9%). However, only a small proportion reported taking regular actions to control salt (10% and 2.6%), and their actual salt consumption was much higher than the recommended level of ≤5 g/day (98% of the participants consumed ≥5 g salt/day in the present study). Our results of participants’ levels of knowledge and attitudes are comparable to those reported from studies in India [22], China [23], Malaysia [24], the United Arab Emirates [25], Australia [26], Samoa [27], Ethiopia [28], and the five sentinel countries of the Americas [29]; however, the proportion of our participants taking regular actions to control their salt intake was comparatively lower compared with those studies.

Similar to our result, studies from China [23], Jordan [30], and Malaysia [31] also reported that self-perceived salt consumption was significantly associated with 24 h urinary salt excretion. A study from India reported that salt-related behaviours such as refraining from adding salt while cooking and avoiding eating processed foods and pickles were inversely associated with 24 h urinary salt excretion [22]. A study conducted in China found that knowledge, i.e., knowing the risk factor of hypertension, was inversely associated with 24 h salt excretion [23], while another study from China concluded that salt-related knowledge, behaviours, and overall KAB scores were all inversely associated with 24 h salt excretion [32]. It is worth noting that the KAB assessment questionnaire/tools used in the Chinese studies differed from those used in our study. The studies from Samoa [27], Australia [26], and Malaysia [24], on the other hand, reported no association between KAB and 24 h urinary salt excretion using a similar questionnaire/tool.

The present study determined that 81% of participants regarded their salt intake as normal (55%) or very low/low (26%). However, analysis of the 24 h urinary assessments revealed that almost all participants (98%) exceeded the recommended daily salt intake of <5 g. This disparity between self-perceived and actual salt consumption suggests a lack of awareness among participants regarding their salt intake. Similar findings were reported in previous studies [21,23,24,26,27], indicating a tendency for individuals to underestimate their salt consumption, which can result in dietary misconceptions [33] and lead to fewer people adopting salt reduction measures. Indeed, there was a positive correlation between self-perceived salt consumption and 24 h salt excretion, behaviours of adding extra salt, and processed food consumption in the regression analyses. On the other hand, those with a self-perception of lower salt consumption were significantly more likely to take actions to control salt intake. These results suggest that participants’ self-perception of salt intake may influence both their actual salt consumption and their readiness to adopt salt reduction measures. Therefore, targeted interventions are necessary to increase awareness among high-risk groups and promote a reduction in salt intake when individuals perceive that they consume an excessive quantity.

People should be aware of the harmful effects of excessive salt intake, as well as the major dietary sources of sodium and the maximum daily salt recommendation intake. Understanding these facts can help individuals make informed decisions about their salt intake and motivate them to adopt salt-reduction behaviours. Health Belief models (HBMs) postulate that when people are fully aware/knowledgeable of the causality of disease conditions, the severity and harmful effects of certain behaviours, and the benefit of preventive measures, they are more likely to take preventive actions [34]. The association between salt-related knowledge and the intention and actions taken to control salt intake has been reported in previous studies [12,35,36]. Our result, however, did not find such an association in the regression analysis. In contrast to other studies that included multiple questions to assess salt-related knowledge [12,36], we included only one question, which may not have been enough to capture the participants’ comprehensive knowledge related to salt and may have resulted in a non-significant association.

Previous studies showed that counselling from health professionals on dietary salt restriction was an effective way to adopt healthier behaviours such as practising actions to control salt intake through acquiring knowledge, skills, and behaviour modification [35], which resulted in lower SBP and control BP among participants [37]. This was also evident from our study, where participants who were counselled by health professionals to reduce dietary salt had a higher likelihood of taking actions to control salt compared with those who did not receive counselling (OR = 6.89; 95% CI: 2.50, 19.01, *p* < 0.001). However, it should be noted that two-thirds of the participants (63%) who received counselling from a health professional to reduce dietary salt (Table 3) had not practised such behaviours, which may reflect the lack of motivation to initiate actions among participants or a lack of behavioural knowledge of salt control techniques, resulting in a gap between knowledge, attitudes, and actual behaviours. Therefore, comprehensive knowledge/information regarding salt and sodium and their relationship, sources of salt in food, detrimental health effects of excessive salt intake, and skills including appropriate salt control techniques such as reading sodium labels, the appropriate use of salt-restricted spoons, salt substitutes, and salt-restriction diets should be comprehensively provided. Further, motivation-oriented activities should be incorporated into the interventions as behaviour-changing techniques [38].

Studies suggest that salt-related knowledge and attitudes are modifiable and mediating factors that influence the behaviour of discretionary salt use [39]. Unlike Western countries where manufactured processed foods are abundant [40], in Nepal, discretionary salt use during cooking is a ubiquitous practice [41]. High discretionary salt use means that people have more individual control over their salt intake, which supports population-level interventions such as mass media mobilization and public health campaigns to become more effective, in addition to policy-level interventions such as taxation of food high in sodium, front-of-pack labelling regulation, and reformulation targets for processed/packaged food (voluntary or mandatory). Salt-restriction tools (such as calibrated salt-restricted spoons), salt substitutes (low-sodium salt and/or potassium-added salt), and health education were found to be relevant and effective in lowering salt consumption in settings where the prime source of salt is discretionary at home [8,42,43]. Therefore, the promotion of these initiatives can be an appealing strategy for salt reduction intervention in Nepal. However, evidence suggests that compared with standalone interventions, multifaceted interventions encompassing both policy-level structural changes and community-level behaviour changes have been effective in lowering salt intake by addressing both the sources of salt, such as processed foods, and discretionary use [8,44,45,46].

In addition, the promotion of dietary potassium intake should be integrated into salt-reduction programs and interventions, as both high sodium intake and low potassium intake are associated with hypertension and an increased risk of CVDs [47]. The WHO recommends a daily potassium intake of at least 90 mmol (3.51 g) for adults [47]. However, our results indicated that participants had an average potassium intake of 49.76 mmol/day (1.94 g/day), which is significantly below the recommended level. Additionally, the sodium-to-potassium ratio among participants was 5.04, which is considerably higher than the WHO’s recommended ratio of <1 [47]. This elevated ratio underscores the need for dietary interventions focused on reducing sodium intake while increasing potassium intake to align with the WHO guidelines and improve overall health outcomes. Therefore, raising awareness about the major food sources of potassium and encouraging their consumption is crucial for health improvement.

Several community-based interventions led by community health workers or community volunteers have resulted in lower salt intake and BP in discretional salt use settings [11,48,49]. The interventions were based on lifestyle modification and behavioural change communications such as health messages on the harmful effects of high salt and saturated fat intake, smoking, and alcohol consumption; promotion of physical activities and consumption of green vegetables and fruits; BP screening and monitoring with digital sphygmomanometer; and referral [11,48,49]. A recent cluster-randomized trial conducted in Nepal also showed that Female Community Health Volunteer (FCHV)-led lifestyle interventions and BP screening activities resulted in a lower mean SBP in an intervention group compared with a control [50]. Furthermore, females are mainly responsible for food shopping, handling, and preparation in Nepal. They are considered gatekeepers to food and health in the household; therefore, targeting females with awareness and behaviour modification programs will possibly result in decreased salt consumption and improve the overall health of a household. A successful salt reduction program in the U.K., for instance, targeted females for an awareness campaign [51]. FCHVs can play a vital role in facilitating such interventions through existing mothers’ group and women’s group committees. Future studies should include the feasibility, capacity of FCHVs to render the services, scalability, and sustainability of such community-based interventions.

This is to our knowledge the first study in Nepal that compared the association between salt-related KAB and salt excretion using a 24 h urinary method that allows for precise comparisons with actual salt intake. Our study has some potential limitations. Being a cross-sectional study, there was some uncertainty in establishing a causal relationship between exposures and outcomes, although our findings were aligned with previous literature in this field. The use of the WHO STEPS instrument, which included only one question related to knowledge, may limit the comprehensiveness of the results in capturing the salt-related knowledge of the participants. Additionally, we did not measure diuretic use by the participants, which might have led to different results. Another limitation pertains to the geographic scope, as this study was conducted exclusively within a single region of the country. Consequently, the generalizability of its findings to other regions may be limited.

The findings of this study have important public health implications in Nepal. People at high risk of consuming higher salt and less likely to adopt salt reduction measures, as identified in this study, can be prioritized for a focused intervention. While no association was found between salt-related knowledge and behaviours and 24 h urinary salt excretion, future studies are recommended with a comprehensive KAB questionnaire, which may produce more evidence. The present study found significant associations in the effectiveness of health professionals in dietary counselling for salt restriction. These findings suggest an opportunity to similarly evaluate the effectiveness of community-driven awareness and education program involving FCHV engagement. Furthermore, this study provides theory-based insights for the future design of a national salt-reduction programs and interventions in Nepal.

## 5. Conclusions

The mean population salt intake in Nepal exceeds the WHO recommendation [1], but the country lacks specific salt-reduction programs. Although participants have basic knowledge of the negative effects of excessive salt intake and favourable attitudes towards reducing it, they have not taken actions, indicating a gap between knowledge, attitude, and actual behaviours. To bridge this gap, programs should include health messaging, behavioural change communication, and motivational interventions to translate intention into action. Nepal urgently needs tailored national salt reduction programs incorporating both policy and community-level interventions to achieve a 30% relative reduction in the mean population salt intake by 2025 and consequently possibly alleviate the burden of CVDs. While community-based interventions led by community health workers/volunteers have been effective in some low-income settings, more information is needed on the structural and social barriers and facilitators for sodium reduction in Nepal.

## Figures and Tables

**Table 1:** Socio-demographic characteristics.

Characteristics	Mean or (*n*)	SD or (%)
Age (years)	49.60	9.82
Weight (kg)	63.70	11.59
Height (cm)	156.03	(8.2
BMI (kg/m^2^)	26.15	428
Systolic blood pressure (mm Hg)	129.06	18.03
Diastolic blood pressure (mm Hg)	83.37	10.26
Hypertension	(178)	(39.47)
Diabetes (self-reported)	(45)	(10.90)
**24 h urine**		
Sodium (g/day)	5.31	1.89
Potassium (g/day)	1.94	0.77
Creatinine (mg/day)	1232.77	524.94
Sodium-to-potassium ratio	5.04	2.04
Urine volume (mL/day)	2584.03	1100.14
Salt excretion, g/day	13.28	4.72
Proportion with ≥5 g/day	(441)	(97.78)

SD, standard deviation; BMI, body mass index (includes missing values, n = 1).

**Table 2:** Association between socio-demographic characteristics and salt-related knowledge and attitudes.

Characteristics	Total, n (%)	Mean (±SD) Salt Intake (g/day) *	Proportion of Participants with Salt-Related Knowledge and Attitudes (%) *						
			Knowledge		Attitudes				
			High Salt Intake Causes Health Problems		Self-Perceived Salt Intake			Importance of Lowering Salt Intake	
			Yes	No ^†^	Extremely High/ High	Normal	Very Low/ Low	Very Important/ Somewhat Important	Not Important ^¶^
**Age, years**									
18–44	153 (33.92)	14.34 (4.91)	86.93	13.07	22.88	57.52	19.61	86.93	13.07
45–70	298 (66.08)	12.73 (4.53)	80.87	19.13	17.11	53.69	29.19	86.91	13.09
*p*-value		**<0.01**	0.10		0.06			0.10	
**Sex**									
Male	156 (34.59)	14.40 (5.06)	84.62	15.38	14.74	50	35.26	89.10	10.90
Female	295 (65.41)	12.69 (4.43)	82.03	17.97	21.36	57.63	21.02	85.76	14.24
*p*-value		**<0.01**	0.49		**<0.01**			0.32	
**Ethnicity**									
Dalit	51 (11.31)	12.90 (5.45)	76.47	23.53	19.61	45.10	35.29	84.31	15.69
Disadvantaged caste	67 (14.86)	13.12 (5.04)	80.60	19.40	22.39	62.69	14.93	83.58	16.42
Upper caste	333 (73.84)	13.37 (4.54)	84.38	15.62	18.32	54.95	26.73	87.99	12.01
*p*-value		0.76	0.32		0.13			0.52	
**Highest education**									
Primary level	265 (58.76)	12.70 (4.74)	79.62	20.38	20	56.98	23.02	85.28	14.72
Higher secondary level	158 (35.03)	14.12 (4.78)	87.97	12.03	18.99	53.16	27.85	88.61	11.39
University level	28 (6.21)	14.08 (3.16)	85.71	14.29	10.71	46.43	42.86	92.86	7.14
*p*-value		**<0.01**	0.08		0.20			0.45	
**BMI**									
Underweight	17 (3.78)	12.64 (4.26)	82.35	17.65	11.76	47.06	41.18	88.24	11.76
Normal	159 (35.33)	12.82 (4.50)	82.39	17.61	20.13	57.86	22.01	86.16	13.84
Overweight	192 (42.67)	13.42 (4.61)	84.90	15.10	17.19	52.60	30.21	89.58	10.42
Obese	82 (18.22)	13.86 (5.34)	80.49	19.51	23.17	56.10	20.73	82.93	17.07
*p*-value		0.36	0.79		0.34			0.47	
**Family size**									
Small (≤4 people)	291 (64.52)	13.02 (4.67)	82.13	17.87	19.93	55.67	24.40	85.22	14.78
Large (>4 people)	160 (35.48)	13.76 (4.78)	84.38	15.63	17.50	53.75	28.75	90	10
*p*-value		0.11	0.54		0.57			0.15	
**HTN status**									
Normotensive	104 (23.06)	13.39 (4.81)	74.04	25.96	24.04	61.54	14.42	79.81	20.19
Pre-hypertensive	169 (37.47)	13.17 (4.59)	84.62	15.38	18.93	57.99	23.08	88.76	11.24
Hypertensive	178 (39.47)	13.32 (4.81)	86.52	13.48	16.29	48.31	35.39	89.33	10.67
*p*-value		0.92	**<0.05**		**<0.05**			0.05	
**Counsel to reduce dietary salt**									
Yes	81 (17.96)	13.20 (5.08)	92.59	7.41	8.64	43.21	48.15	95.06	4.94
No	370 (82.04)	13.30 (4.64)	80.81	19.19	21.35	57.57	21.08	85.14	14.86
*p*-value		0.87	**<0.05**		**<0.001**			**<0.05**	
**History of elevated BP**									
Yes	118 (26.16)	13.25 (4.87)	91.53	8.47	11.86	44.07	44.07	93.22	6.78
No	333 (73.84)	13.29 (4.67)	79.88	20.12	21.62	58.86	19.52	84.68	15.32
*p*-value		0.94	**<0.01**		**<0.001**			**<0.05**	
**Anti-HTN drug use in the past 2 weeks**									
Yes	89 (19.73)	12.99 (4.42)	93.26	6.74	8.99	43.82	47.19	95.51	4.49
No	362 (80.27)	13.35 (4.79)	80.39	19.61	21.55	57.73	20.72	84.81	15.19
*p*-value		0.5120	**<0.01**		**<0.001**			**<0.01**	
**History of diabetes**									
Yes	45 (10.90)	13.71 (3.82)	91.11	8.89	11.11	43.90	44.44	93.33	6.67
No	368 (89.10)	13.27 (4.74)	82.61	20.11	19.76	55.16	24.73	86.41	13.59
*p*-value		0.55	0.20		**<0.05**			0.24	

BMI, body mass index (includes missing values, n = 1); HTN, hypertension; SD, standard deviation. ^¶^ not important category includes the “do not know” response (n = 1); ^†^ No category includes the “do not know” response (n = 2); * *p*-values for the differences in 24 h salt intake and salt-related knowledge and attitudes by socio-demographic characteristics were obtained using analysis of variance (ANOVA) and Chi-square tests (or Fisher’s exact tests as appropriate), respectively (bolded numbers represent significant *p*-value as indicated).

**Table 3:** Association between socio-demographic characteristics and salt-related behaviours.

Characteristics	Proportion of Participants with Salt-Related Behaviours (%) *							
	Add Extra Salt			Processed Food Consumption			Take Actions to Control Salt	
	Always/Often	Sometimes	Rarely/Never	Always/Often	Sometimes	Rarely/Never	Yes	No
**Age, years**								
18–44	6.54	19.61	73.86	5.23	64.05	30.72	6.54	93.46
45–70	3.36	17.79	78.86	2.35	44.63	53.02	11.41	88.59
*p*-value	0.25			**<0.001**			0.10	
**Sex**								
Male	2.56	9.62	87.82	2.56	45.51	51.92	16.67	83.33
Female	5.42	23.05	71.53	3.73	54.24	42.03	6.10	93.90
*p*-value	**<0.001**			0.13			**<0.001**	
**Ethnicity**								
Dalit	7.84	19.61	72.55	1.96	54.90	43.14	15.69	84.31
Disadvantaged caste	2.99	28.36	68.66	8.96	59.70	31.34	10.45	89.55
Upper caste	4.20	16.22	79.58	2.40	48.95	48.65	8.71	91.29
*p*-value	0.12			**<0.05**			0.29	
**Highest education**								
Primary level	4.91	20.38	74.72	3.40	49.06	47.55	9.43	90.57
Higher secondary level	3.80	17.72	78.48	3.80	56.33	39.87	10.13	89.87
University level	3.57	3.57	92.86	0	42.86	57.14	10.71	89.29
*p*-value	0.20			0.37			0.90	
**BMI**								
Underweight	0	17.65	82.35	0	29.41	70.59	23.53	76.47
Normal	4.40	20.75	74.84	1.89	52.83	45.28	8.18	91.82
Overweight	3.65	18.23	78.13	3.13	52.08	44.79	11.46	88.54
Obese	7.32	14.63	78.05	7.32	51.22	41.46	6.10	93.90
*p*-value	0.77			0.18			0.11	
**Family size**								
Small (≤ four people)	4.12	19.59	76.29	4.47	46.74	48.80	9.62	90.38
Large (>four people)	5	16.25	78.75	1.25	59.38	39.38	10	90
*p*-value	0.66			**<0.05**			0.90	
**HTN status**								
Normotensive	6.73	19.23	74.04	1.92	60.58	37.50	1.92	98.08
Pre-hypertensive	4.14	21.89	73.96	4.14	54.44	41.42	8.28	91.72
Hypertensive	3.37	14.61	82.02	3.37	42.70	53.93	9.76	90.24
*p*-value	0.26			**<0.05**			**<0.001**	
**Counsel to reduce dietary salt**								
Yes	3.70	9.88	86.42	2.47	37.04	60.49	37.04	62.96
No	4.59	20.27	75.14	3.51	54.32	42.16	3.78	96.22
*p*-value	0.07			**<0.05**			**<0.001**	
History of elevated BP								
Yes	2.54	12.71	84.75	4.24	40.68	55.08	26.27	73.73
No	5.11	20.42	74.47	3	54.95	42.04	3.90	96.10
*p*-value	0.08			**<0.05**			**<0.001**	
**Anti-HTN drug use in past 2 weeks**								
Yes	3.37	10.11	86.52	2.25	39.33	58.43	26.97	73.03
No	4.70	20.44	74.86	3.59	54.14	42.27	5.52	94.48
*p*-value	0.054			**<0.05**			**<0.001**	
**History of diabetes**								
Yes	0	17.78	82.22	2.22	37.78	60	22.22	77.78
No	4.62	17.93	77.45	3.80	54.08	42.12	8.15	91.85
*p*-value	0.44			0.07			**<0.01**	

BMI, body mass index (includes missing values, n = 1); HTN, hypertension. * *p*-values for the differences in salt-related behaviours by socio-demographic characteristics were obtained using Chi-square tests (or Fisher’s exact tests as appropriate) (bolded numbers represent significant *p*-values as indicated).

**Table 4:** Association between salt-related knowledge and attitudes and salt-related behaviours.

Salt-Related Knowledge and Attitudes	Salt-Related Behaviours ^†^					
	Adding Extra Salt Always ^a^		Consuming Processed Food Always ^a^		Taking Actions to Control Salt ^¥^	
	AOR ^†^	95% CI	AOR ^†^	95% CI	AOR ^†^	95% CI
**High salt intake causes health problems**						
No ^b^	1.00 (Ref.)					
Yes	0.60	0.33, 1.09	1.73	0.99, 3.02	3.19	0.65, 15.70
**Self-perceived salt consumption ^¥^**						
Normal	1.00 (Ref.)					
Very low/low	0.47	0.22, 1.01	0.60	0.36, 0.98 *	4.22	1.90, 9.37 ***
Extremely high/high ^c^	3.59	2.03, 6.33 ***	1.90	1.06, 3.40 *	0.14	0.01, 2.58
**Importance of lowering salt intake**						
Not important ^d^	1.00 (Ref.)					
Very Important/somewhat important	1.06	0.53, 2.14	1.61	0.87, 2.97	5.44	0.60, 49.19

95% CI, 95% confidence interval; AOR, adjusted odds ratio; HTN, hypertension; Ref., reference category. ^a^ Always, often, and sometimes merged. ^b^ No category includes the “do not know” response (n = 2). ^c^ Extremely high, high, and normal merged. ^d^ Not important category includes the “do not know” response (n = 1). ^†^ Model adjusted for age sex, caste, family size, hypertension status, counselled by a health professional to reduce salt intake, history of elevated blood pressure, antihypertensive drug use in the past 2 weeks, and history of diabetes; ^¥^ Penalized likelihood regression was used among these variables because of complete separation; * *p* < 0.05, *** *p* < 0.001.

**Table 5:** Association between salt-related knowledge, attitudes, and behaviours and 24 h urinary salt excretion.

Salt-Related KAB	n (%)	Adjusted Estimate ^†^		
		Mean Salt Intake (g/day)	95% CI	*p*-Value
**Knowledge**				
***High salt intake causes health problems***				
Yes	374 (82.93)	13.28	12.82, 13.75	0.78
No ^a^	77 (17.07)	13.14	12.11, 14.17	
**Attitudes**				
***Self-perceived salt consumption***				
Extremely high/high	86 (19.07)	14.42	13.45, 15.39	0.03 *
Normal	248 (54.99)	13.11	12.54, 13.67	
Very low/low	117 (25.94)	12.73	11.89, 13.65	
***Importance of lowering salt intake***				
Very important/somewhat important	392 (86.92)	13.33	12.87, 13.78	0.42
Not important ^b^	59 (13.08)	12.81	11.63, 13.99	
**Behaviours**				
***Add extra salt***				
Always/often	20 (4.43)	14.67	12.66, 16.68	0.36
Sometimes	83 (18.40)	13.00	12.31, 14.30	
Rarely/never	348 (77.16)	13.17	12.69, 13.65	
***Processed food consumption***				
Always/often	15 (3.33)	14.57	12.23, 16.91	0.53
Sometimes	231 (51.22)	13.23	12.64, 13.83	
Rarely/never	205 (45.45)	13.19	12.55, 13.83	
***Take actions to control salt intake***				
Yes	44 (9.76)	13.51	12.15, 14.89	0.70
No	407 (90.24)	13.23	12.79, 13.67	
**If yes, what are they** **^c^** **(n = 44)?**				
***Avoid eating processed foods***				
Yes	14 (31.82)	13.88	10.37, 17.39	0.88
No	30 (68.18)	13.53	11.30, 15.76	
***Check salt/sodium labelling on food***				
Yes	11 (25)	15.61	11.83, 19.39	0.25
No	33 (75)	12.99	10.97, 15.01	
***Buy low-salt/sodium alternatives***				
Yes	1 (2.27)	12.72	0.60, 24.85	0.88
No	43 (97.73)	13.67	11.94, 15.40	
***Avoid out-of-home dinning***				
Yes	9 (20.45)	16.25	12.06, 20.43	0.18
No	35 (79.55)	12.98	11.04, 14.91	
***Avoid adding extra salt***				
Yes	36 (81.82)	14.10	12.18, 16.02	0.33
No	8 (18.18)	11.60	7.09, 16.10	
***Use small spoon***				
Yes	10 (22.73)	15.83	11.93, 19.73	0.22
No	34 (77.27)	13.00	11.03, 14.97	
***Apply other measures***				
Yes	9 (20.45)	11.76	7.65, 15.86	0.31
No	35 (79.55)	14.13	12.19, 16.07	

95% CI, 95% confidence interval; KAB, knowledge, attitudes, and behaviours. ^a^ No category includes the “do not know” response (n = 2); ^b^ Not important category includes the “do not know” response (n = 1); ^c^ Participants could take more than one action to control salt intake; ^†^ Model adjusted for possible confounding effects of age, sex, education, and body mass index. * *p* < 0.05.

## Data Availability

The data that support the findings of this study are not publicly available due to privacy concerns but will be made available by the authors upon reasonable request. (specify the reason for the restriction).

## Supplementary Materials

The following supporting information can be downloaded at https://www.mdpi.com/article/10.3390/nu16121928/s1, Table S1: Salt-related knowledge, attitudes, and behaviour questionnaire included in this study; Table S2: Association between socio-demographic characteristics and salt-related behaviours; Figure S1: Post hoc analyses between adding extra salt always and self-perceived salt consumption (group comparisons); Figure S2: Post hoc analyses between taking actions to control salt and self-perceived salt consumption (group comparisons); Figure S3: Post hoc analyses between 24 h urinary salt excretion and self-perceived salt consumption (group comparisons).
