# Supplementary material for: Association between Salt-Related Knowledge, Attitudes, and Behaviours and 24 h Urinary Salt Excretion in Nepal
Source: Nutrients. 2024 Jun 18;16(12):1928. doi: 10.3390/nu16121928 (PMC11206565; doi:10.3390/nu16121928)
Supplement: Supplementary file 1 [file nutrients-16-01928-s001.zip › S1_Figure 1.pdf]

Supplemental Figure S1. Post-hoc analyses between adding extra salt always and self-perceived salt consumption (group comparisons)

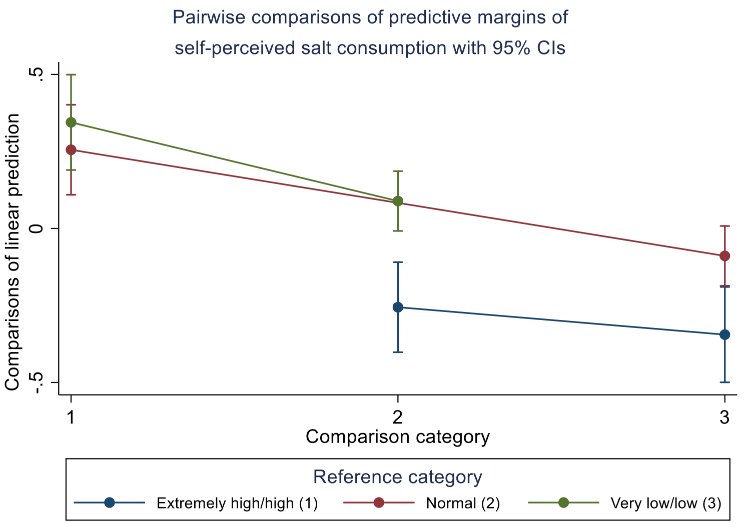

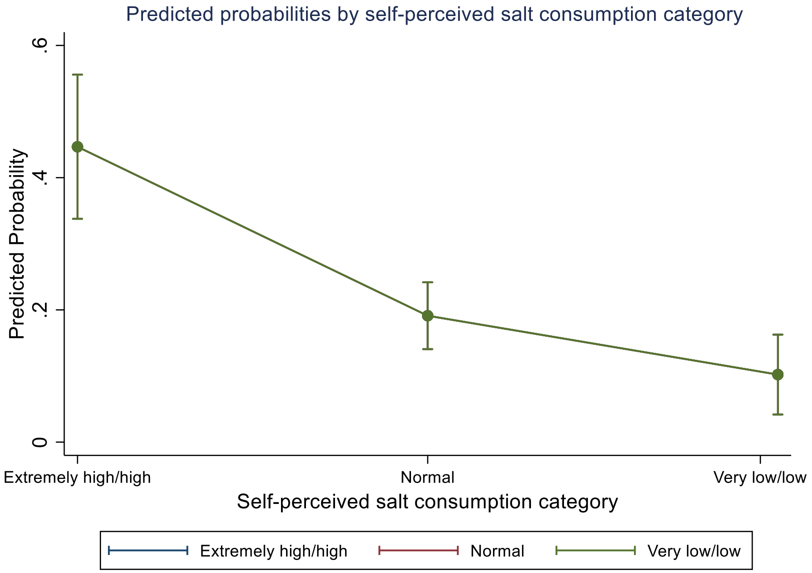

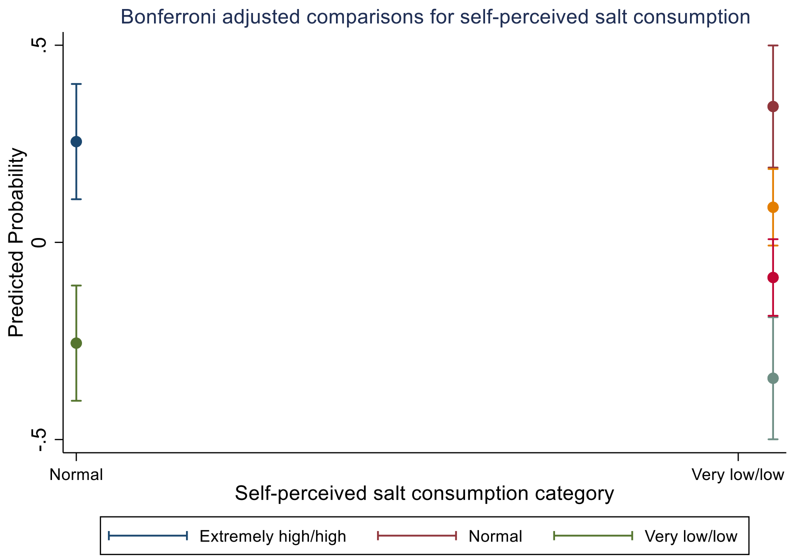


```
. margins salt_eat2, pwcompare(effects) mcompare(bonferroni)

Pairwise comparisons of predictive margins               Number of obs = 413
Model VCE: OIM

Expression: Pr(add_salt_alway_often_sometimes), predict()

---------------------------
                |  Number of
                | comparisons
----------------+----------
      salt_eat2 |          3
---------------------------

-----------------------------------------------------------------------------------------------
                                    |            Delta-method    Bonferroni         Bonferroni
                                    |  Contrast   std. err.      z    P>|z|    [95% conf. interval]
------------------------------------+----------------------------------------------------------
                          salt_eat2 |
      Normal vs Extremely.High.&.High | -.2555554   .0610529    -4.19  0.000    -.4017148   -.109396
Low.&.Very.low vs Extremely.High.&.High | -.3445871   .0646774    -5.33  0.000    -.4994236  -.1897507
           Low.&.Very.low vs Normal | -.0890317    .040559    -2.20  0.084    -.1861291   .0080657
-----------------------------------------------------------------------------------------------
```
