# Supplementary material for: Association between Salt-Related Knowledge, Attitudes, and Behaviours and 24 h Urinary Salt Excretion in Nepal
Source: Nutrients. 2024 Jun 18;16(12):1928. doi: 10.3390/nu16121928 (PMC11206565; doi:10.3390/nu16121928)
Supplement: Supplementary file 1 [file nutrients-16-01928-s001.zip › S1_Figure 2.pdf]

Supplemental Figure S2. Post-hoc analyses between taking actions to control salt and the self-perceived salt consumption (group comparisons)

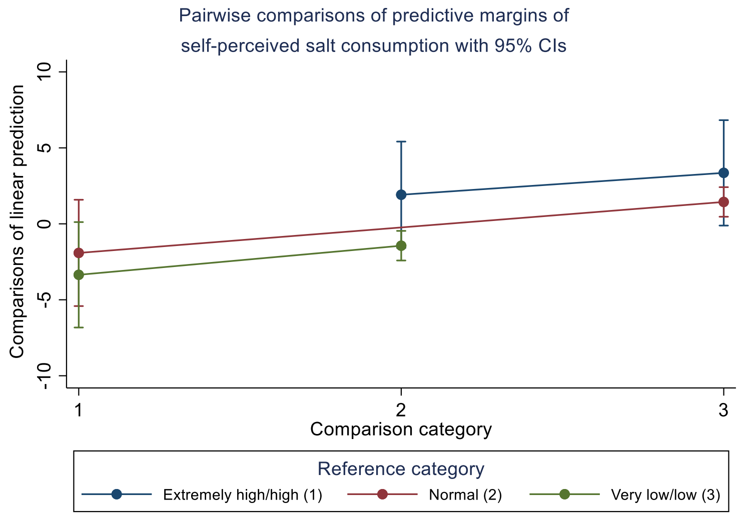

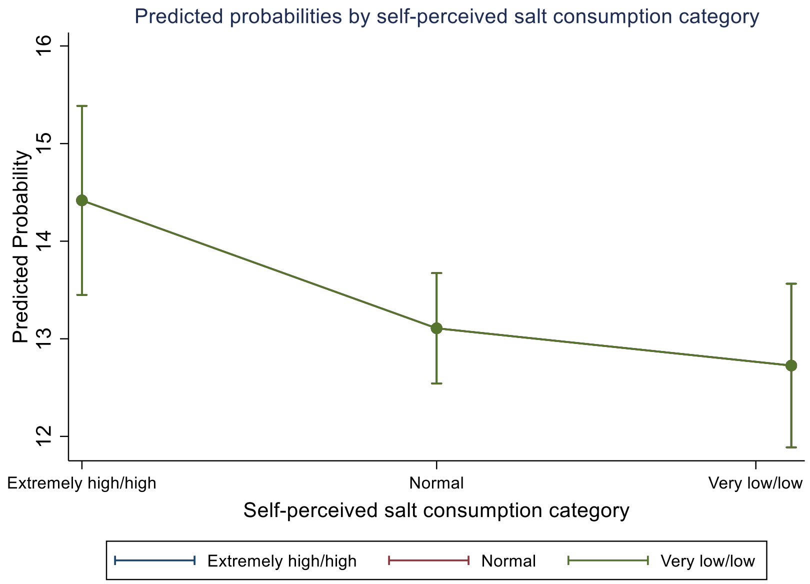

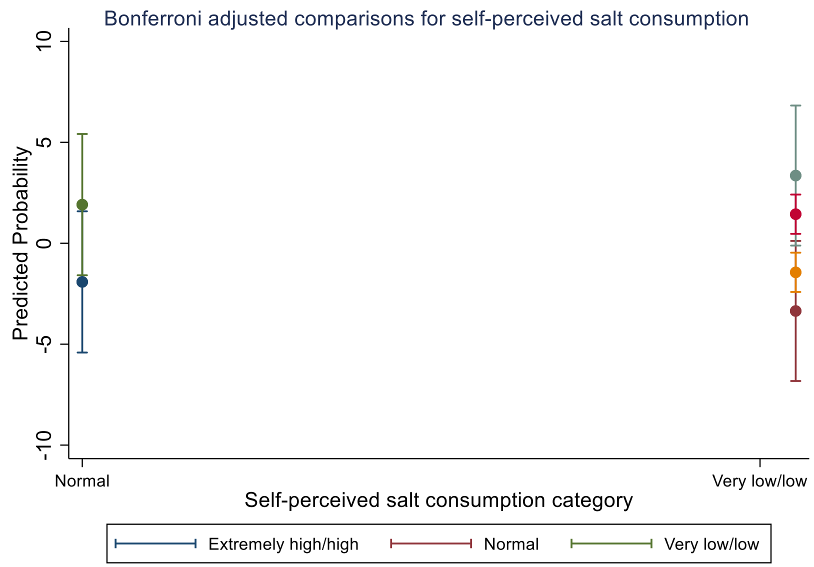


```
. margins salt_eat2, pwcompare(effects) mcompare(bonferroni)

Pairwise comparisons of predictive margins                Number of obs = 413
Model VCE: OIM

Expression: Linear prediction, predict()
```

	Number of comparisons
salt_eat2	3

	Contrast	Delta-method std. err.	Bonferroni z	Bonferroni P>|z|	Bonferroni [95% conf. interval]	
salt_eat2						
Normal vs Extremely.High.&.High	1.914682	1.461423	1.31	0.570	-1.583935	5.4133
Low.&.Very.low vs Extremely.High.&.High	3.354809	1.449372	2.31	0.062	-.1149573	6.824576
Low.&.Very.low vs Normal	1.440127	.4066789	3.54	0.001	.4665461	2.413708
