# Supplementary material for: Association between Salt-Related Knowledge, Attitudes, and Behaviours and 24 h Urinary Salt Excretion in Nepal
Source: Nutrients. 2024 Jun 18;16(12):1928. doi: 10.3390/nu16121928 (PMC11206565; doi:10.3390/nu16121928)
Supplement: Supplementary file 1 [file nutrients-16-01928-s001.zip › S1_Figure 3.pdf]

Supplemental Figure S3. Post-hoc analyses between 24-h urinary salt excretion and the self-perceived salt consumption (group comparisons)

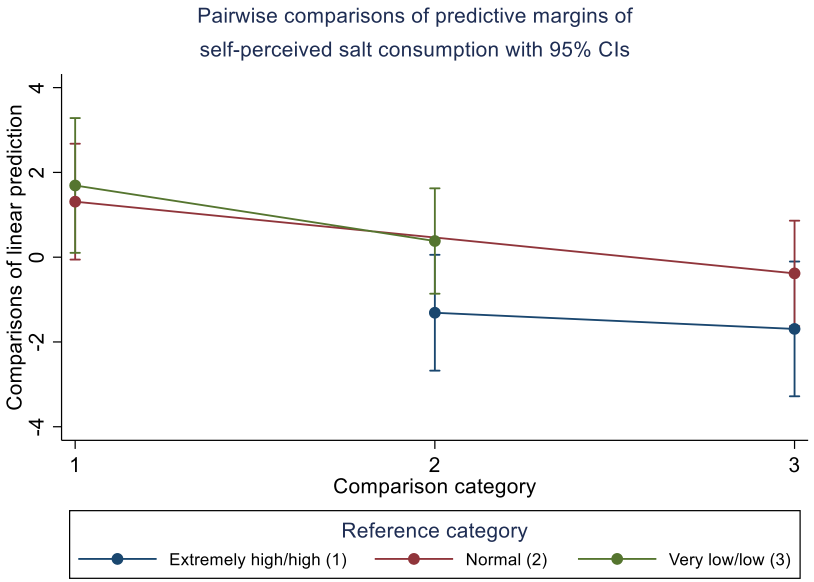

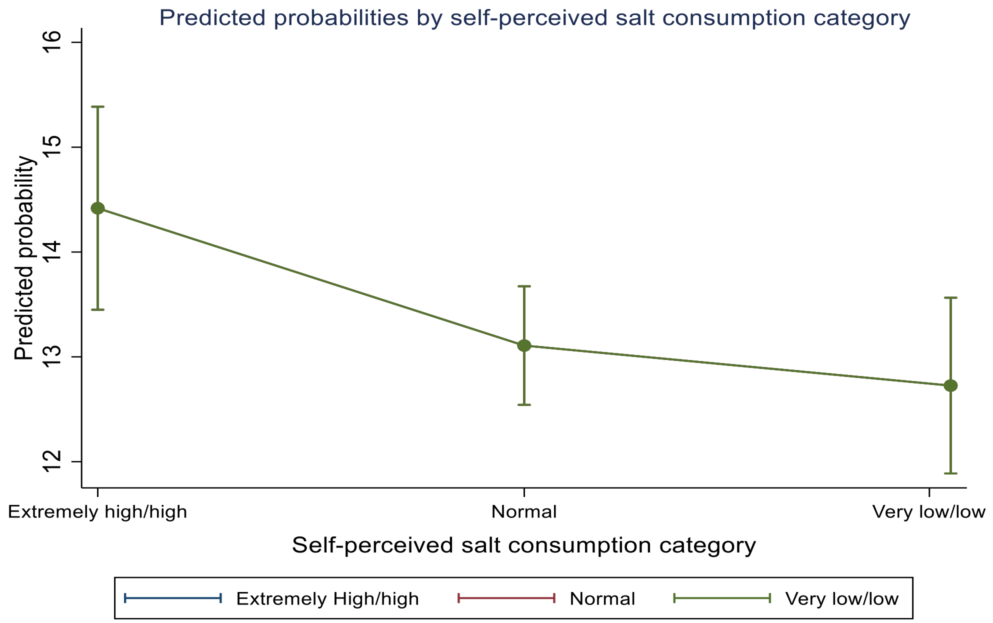

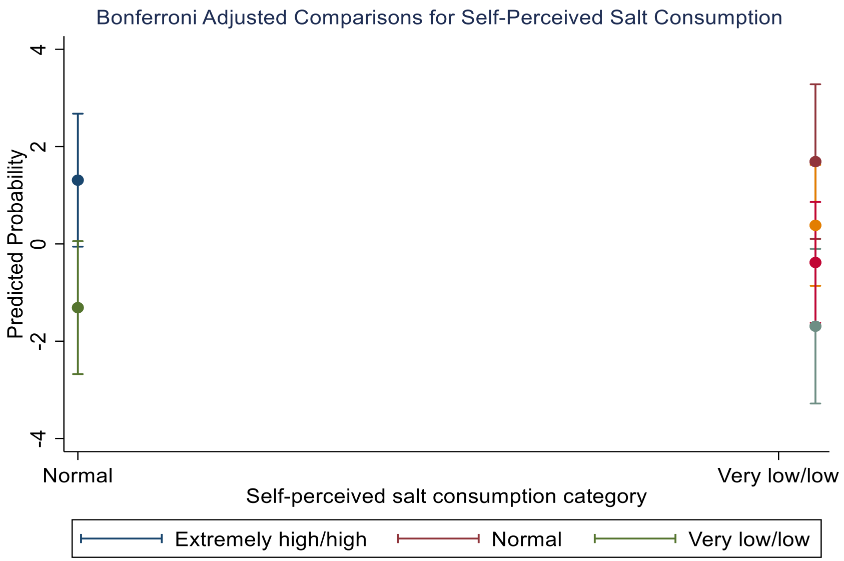


```
. margins salt_eat2, pwcompare(effects) mcompare(bonferroni)

Pairwise comparisons of predictive margins              Number of obs = 450

Expression: Linear prediction, predict()
```

	Number of comparisons
salt_eat2	3

	Contrast	Delta-method std. err.	Bonferroni t	Bonferroni P>|t|	Bonferroni [95% conf.	interval]
salt_eat2						
Normal vs Extremely.High.&.High	-1.310283	.5687279	-2.30	0.065	-2.677008	.0564429
Low.&.Very.low vs Extremely.High.&.High	-1.691964	.6614818	-2.56	0.033	-3.281589	-.1023389
Low.&.Very.low vs Normal	-.3816813	.517258	-0.74	1.000	-1.624718	.8613556
