# Supplementary material for: Association between Salt-Related Knowledge, Attitudes, and Behaviours and 24 h Urinary Salt Excretion in Nepal
Source: Nutrients. 2024 Jun 18;16(12):1928. doi: 10.3390/nu16121928 (PMC11206565; doi:10.3390/nu16121928)
Supplement: Supplementary file 1 [file nutrients-16-01928-s001.zip › S1_Table 1.pdf]

Supplemental Table S1. Salt-related knowledge, attitudes, and behaviours questionnaire included in the study

Salt related KAB	Coded as	Response options	Modified response options after merging[a]
**Knowledge**			
Do you think eating too much salt is bad for your health?	High salt intake causes health problems	• Yes<br>• No<br>• Don't know	• Yes<br>• No/Don't Know
**Attitudes**			
How much salt do you think you consume?	Self-perceived salt intake	• Extremely high<br>• High<br>• Normal (just the right amount)<br>• Low<br>• Very low	• Extremely high/High<br>• Normal (just the right amount)<br>• Very low/Low
In your opinion, how much important to you is lowering salt in your food?	Importance of lowering salt intake	• Very important<br>• Somewhat important<br>• Not important<br>• Do not know	• Very important/Somewhat important<br>• Not important/Don't know
**Behaviours**			
How often do you add salt or a salty sauce such as soy sauce to your food right before you eat it or as you are eating it[b]?	Add extra salt	• Always<br>• Often<br>• Sometimes<br>• Rarely<br>• Never	• Always/Often<br>• Sometime<br>• Never/Rarely
How often do you eat processed foods high in salt[c]?	Processed food consumptions	• Always<br>• Often<br>• Sometimes<br>• Rarely<br>• Never	• Always/Often<br>• Sometime<br>• Never/Rarely
Do you take regular action to control your salt intake?	Take actions to control salt intake	• Yes<br>• No	• Yes<br>• No
If yes, what are they? (Multiple answers)			
• Avoid/minimize consumption of processed foods	Avoid eating processed foods	• Yes<br>• No	• Yes<br>• No
• Look for salt (sodium) label in food when buying	Check salt/sodium labelling in food	• Yes<br>• No	• Yes<br>• No
• Buy food with less salt (buy salt alternatives)	Buy low salt/sodium alternatives	• Yes<br>• No	• Yes<br>• No

• Avoid/minimize eating out/out-of-home dining	Avoid out-of-home dinning	• Yes<br>• No	• Yes<br>• No
• Avoid/minimize adding extra salt in food before eating	Avoid adding extra salt	• Yes<br>• No	• Yes<br>• No
• Use small spoon for salt picking	Use small spoon	• Yes<br>• No	• Yes<br>• No
• Other measures to reduce salt	Apply other measures	• Yes<br>• No	• Yes<br>• No

[a]Response options were modified/merged due to low or very low observations in some categories.

[b]In Nepal, the major salt use practice is discretionary, mainly adding salt during meal preparation. Therefore, 'adding extra salt' refers to adding salt at the table to a prepared meal.

[c]Processed foods high in salt collectively refer to packaged salty snacks such as noodles, salty biscuits, chips, pappadum; salty condiments such as pickles, chutney, dried fish; and other processed foods such as cheese, processed meat etc.
