# Supplementary material for: Association between Salt-Related Knowledge, Attitudes, and Behaviours and 24 h Urinary Salt Excretion in Nepal
Source: Nutrients. 2024 Jun 18;16(12):1928. doi: 10.3390/nu16121928 (PMC11206565; doi:10.3390/nu16121928)
Supplement: Supplementary file 1 [file nutrients-16-01928-s001.zip › S1_Table 2.pdf]

Supplemental Table S2. Association of socio-demographic characteristics with salt-related behaviours

Characteristics[a]	Salt-related behaviours[†]					
	Adding extra salt always[b]		Consuming processed food always[b]		Taking actions to control salt	
	AOR†	95% CI	AOR†	95% CI	AOR†	95% CI
**Age group**						
18-44				1.00 (Ref.)		
45-70	0.81	0.45, 1.44	0.48	0.29, 0.79**	0.52	0.18, 1.55
**Sex**						
Male				1.00 (Ref.)		
Female	2.23	1.22, 4.08**	1.20	0.76, 1.87	0.46	0.21, 1.02
**Caste**						
Dalit				1.00 (Ref.)		
Disadvantaged caste	1.11	0.41, 2.97	1.33	0.54, 3.27	5.00	0.98, 25.61
Upper caste	0.75	0.33, 1.72	0.64	0.32, 1.28	1.65	0.47, 5.80
**Family size**						
Small (≤4 people)				1.00 (Ref.)		
Large (>4 people)	0.94	0.54, 1.61	1.58	1.01, 2.49*	0.93	0.40, 2.18
**HTN status**						
Normotensive				1.00 (Ref.)		
Pre-hypertensive	1.18	0.62, 2.26	1.08	0.61, 1.92	2.46	0.49, 12.28
Hypertensive	1.05	0.45, 2.46	0.99	0.48, 2.03	0.82	0.10, 6.59
**Counsel to reduce dietary salt intake**						
No				1.00 (Ref.)		
Yes	0.70	0.23, 1.30	0.75	0.39, 1.43	6.89	2.50, 19.01***
**History of elevated BP**						
No				1.00 (Ref.)		
Yes	0.82	0.24, 2.79	1.31	0.50, 3.43	4.42	0.92, 21.31
**Anti-HTN drugs used in past 2 weeks**						
No				1.00 (Ref.)		
Yes	1.18	0.32, 4.41	0.75	0.28, 2.01	0.96	0.22, 4.14
**History of diabetes**						
No				1.00 (Ref.)		
Yes	1.26	0.51, 3.13	0.74	0.37, 1.50	1.59	0.58, 4.34

95% CI, 95% confidence interval; AOR, adjusted odds ratio; BMI, body mass index (includes missing value, n=1); HTN, hypertension; Ref., reference category.

[a]Socio-demographic variables which were significant at $p<0.05$ in the bivariate analysis were included in the current model; [b]Always, often, and sometimes merged; [†]Model adjusted for age sex, caste, family size, hypertension status, counselled by health professional to reduce salt intake, history of elevated blood pressure, antihypertensive drugs used in the past 2 weeks and history of diabetes.

*p<0.05, **p<0.01, ***p<0.001.
